# Alignment between Chronic Disease Policy and Practice: Case Study at a Primary Care Facility

**DOI:** 10.1371/journal.pone.0105360

**Published:** 2014-08-20

**Authors:** Claire A. Draper, Catherine E. Draper, Graham F. Bresick

**Affiliations:** 1 School of Public Health and Family Medicine, Health Sciences Faculty, University of Cape Town, Cape Town, South Africa; 2 UCT/MRC Research Unit for Exercise Science and Sports Medicine, Sports Science Institute of South Africa, Newlands, South Africa; Toronto Western Hospital, Canada

## Abstract

**Background:**

Chronic disease is by far the leading cause of death worldwide and of increasing concern in low- and middle-income countries, including South Africa, where chronic diseases disproportionately affect the poor living in urban settings. The Provincial Government of the Western Cape (PGWC) has prioritized the management of chronic diseases and has developed a policy and framework (Adult Chronic Disease Management Policy 2009) to guide and improve the prevention and management of chronic diseases at a primary care level. The aim of this study is to assess the alignment of current primary care practices with the PGWC Adult Chronic Disease Management policy.

**Methods:**

One comprehensive primary care facility in a Cape Town health district was used as a case study. Data was collected via semi-structured interviews (n = 10), focus groups (n = 8) and document review. Participants in this study included clinical staff involved in chronic disease management at the facility and at a provincial level. Data previously collected using the Integrated Audit Tool for Chronic Disease Management (part of the PGWC Adult Chronic Disease Management policy) formed the basis of the guide questions used in focus groups and interviews.

**Results:**

The results of this research indicate a significant gap between policy and its implementation to improve and support chronic disease management at this primary care facility. A major factor seems to be poor policy knowledge by clinicians, which contributes to an individual rather than a team approach in the management of chronic disease patients. Poor interaction between facility- and community-based services also emerged. A number of factors were identified that seemed to contribute to poor policy implementation, the majority of which were staff related and ultimately resulted in a decrease in the quality of patient care.

**Conclusions:**

Chronic disease policy implementation needs to be improved in order to support chronic disease management at this facility. It is possible that similar findings and factors are present at other primary care facilities in Cape Town. At a philosophical level, this research highlights the tension between primary health care principles and a diseased-based approach in a primary care setting.

## Introduction

In 2010, the WHO reported that chronic diseases were by far the leading cause of death worldwide and that their impact was steadily increasing [1]. The total number of deaths from chronic diseases was reported to be double that of all infectious diseases (including HIV/AIDS, tuberculosis and malaria), maternal/perinatal conditions, and nutritional deficiencies combined [2]. Eighty percent of chronic disease deaths occur in low- and middle-income countries (LMICs) [1], [3], [4]. A small number of modifiable risk factors are responsible for most chronic diseases, namely unhealthy diet, physical inactivity and tobacco use [2], [5]. Many developed countries have established policies to appropriately manage and prevent chronic diseases [6]–[12]. Similar policies have also been developed in LMICs; however, more policies and plans exist in Latin America and Asia than in Africa.

South Africa, a LMIC, faces a quadruple burden of infectious, chronic, perinatal, and injury-related diseases, present in both rural and urban areas [13]. The WHO estimates that the burden from chronic diseases in South Africa is two to three times higher than in developed countries, disproportionately affecting the poor living in urban and peri-urban settings [13]. The primary health care approach [14] has been proposed as a strategy for improving health in South Africa, and can be applied to the reduction of morbidity and mortality associated with chronic diseases. According to the Declaration of Alma-Ata, primary health care should provide promotive, preventive, curative and rehabilitative services to address the main health problems in communities [14]. The importance of this approach was highlighted again in recent years [15]–[18]. There has also been strong evidence of the benefits of primary care-oriented health systems and primary care is most effective when aligned with the principles of the primary health care approach [19], [20]. By intervening at the primary care level with strategies to reduce modifiable risk factors in communities and ensure early detection and treatment, the burden of chronic diseases on the health care system could be reduced [21]. It is possible that this could impact positively on families and communities, creating opportunities to emphasize family- and community-oriented care.

**Figure 1:**
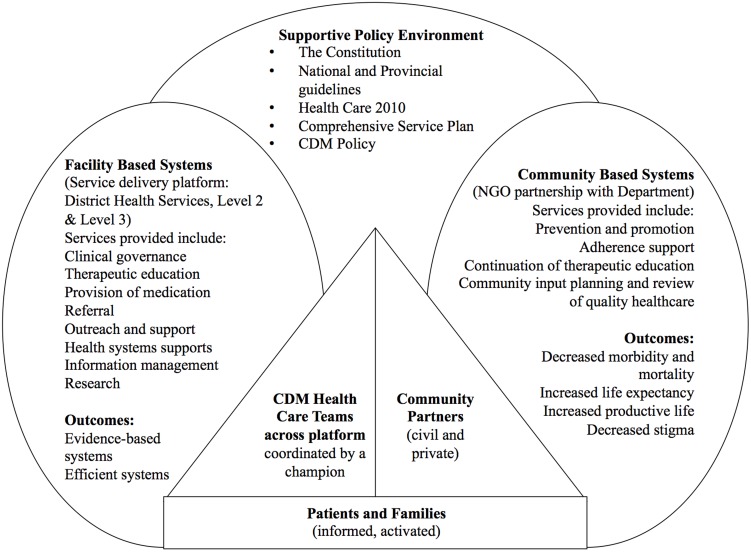
Overarching model for the PGWC Adult Chronic Disease Management Policy [25].

The South African Department of Health has developed national guidelines for the management of various chronic diseases at primary care level, including diabetes, [22] hypertension [23] and asthma [24]. In the Western Cape province of South Africa, management of chronic diseases is one of the key service priorities for the provincial government administration [25]. In 2009, the Provincial Government of the Western Cape (PGWC) developed the Adult Chronic Disease Management Policy (see figure 1 for a summary of the policy) which provides a framework for managing chronic diseases at a primary care level [25]. Using national law and health policies as the overarching guideline, the policy advocates facility-based diagnosis and stabilisation of chronic conditions (co-ordinated by one staff member playing the role of a ‘champion’) alongside community-based prevention, maintenance and support (using community partners such as non-profit organisations - NPOs). It also proposes regular clinical audits (using a specifically developed tool) to assess the quality of care delivered and the attainment of treatment goals [25]. While this tool has gathered valuable data, it only provides part of the picture as far as alignment with this policy framework is concerned, and more in-depth methods are required to complete the picture. Other local audits conducted over the past few years in the Cape Town area show that chronic disease care remains suboptimal [26]–[29].

Current literature highlights some of the difficulties of aligning policy with practice within the South African health care system. Looking at local health policies and practice, Rispel et al. [30] noted elements of progress as well as on going challenges in a number of areas. Another study by Lund et al. [31] revealed that despite progressive policy, significant discrepancies in resources, data collection, services and health needs still existed [31].

Should chronic disease policies be well implemented, they have the potential to make a significant difference to the health of the population served [3]. Since the implementation of the PGWC policy had not been formally evaluated, the aim of this study was to assess the alignment of current primary care practices with the PGWC Adult Chronic Disease Management policy, and identify factors influencing the implementation of the policy and the primary health care approach, focusing on one primary care facility.

## Methods

### Study setting and participants

An exploratory qualitative study was conducted to investigate policy alignment with practice at one specific primary care facility, drawing on qualitative methods to collect data in the form of semi-structured interviews and focus groups. The words ‘case study’ in the title refer to the focus on just one primary care facility rather than indicating a specific case-study design. The comprehensive primary care facility used for this study is a Community Health Center (CHC) serving a population of approximately 30 000 people in an under-resourced area of Cape Town, South Africa. PGWC records indicated that the average number of patients seen at this facility per day in 2011 was approximately 850 patients [32]. This particular facility was selected because the principal investigator (a Family Medicine registrar) had a six-month placement there, and was therefore in an ideal position to conduct the case study and contribute a greater level of understanding to the analysis of the data. Participants in this study were staff employed at the CHC, including facility and clinical managers (n = 3, one Family Physician), doctors (n = 5), nursing staff (n = 5, including clinical nurse practitioners - CNPs) and other allied health professionals (n = 2), as well as health professionals employed at a district or provincial level who support the CHC services (n = 3). All clinical staff members involved with chronic disease management at the facility were invited to participate.

### Interview and focus group guide questions

The guide questions for the interviews and focus groups were developed using the policy document as well as data collected from the Integrated Audit Tool for Chronic Disease Management (a component of the PGWC Chronic Disease Policy). The Audit Tool is used to assess quality of care and evaluates the management of five major chronic diseases: diabetes, hypertension, epilepsy, asthma and chronic obstructive airways diseases (COAD) [25]. Consisting of two components, the audit looks first at the equipment available for chronic disease management at each facility and then involves a folder review for each condition looking at key clinical indicators measured and monitored for each of the five conditions [25]. This tool has been used since 2009 to collect data from this and other similar facilities in the Western Cape. The data collected from the audits conducted at this CHC thus far were collated in order to assess the extent to which processes described in the policy were being implemented as intended. Particular attention was paid to items of the audit where limited information was provided, and more in-depth investigation was deemed necessary. Examples of these items were: details of lifestyle counselling, interface between facility- and community-based services, the Chronic Care Team, therapeutic groups, support groups and health education.

Audit data were analysed for frequencies, and compared across the three years for which data had been collected. Guide questions included questions around policy knowledge, individual role, perceptions of chronic disease management, current practice and perceptions of current practice. These differed slightly between participants depending on their specific roles.

### Data collection

From those clinical staff members who agreed to participate, two focus groups (4 participants per group, n = 8) were conducted, one consisting of doctors and the other of CNPs. The focus group discussions helped inform the purposive selection of other staff for the interviews (n = 10) to further explore pertinent issues. Focus groups and interviews were conducted by the principal investigator (PI), who was a new member of staff at the CHC at the time, and took place at the facility or at the location where the relevant staff member was employed. Each lasted approximately 60–90 minutes. All participants signed written consent which included an emphasis on voluntary participation, freedom to withdraw at any stage with no negative consequences and a guarantee of anonymity.

### Data analysis

All interviews and focus groups were audio recorded and transcribed verbatim by a third party. Interview and focus group texts were coded using the content analytic approach [33] by the PI with input from a co-author using Atlas.ti Qualitative Data Analysis Software (Scientific Software Development GmbH, Berlin, Germany). Themes were identified from the interview and focus group texts and a coding framework was developed using these themes. The structure of the policy document itself also helped in the development of the coding framework. All transcripts were then analysed and the data collated.

Approval for this study was obtained from the PGWC (Department of Health) as well as the University of Cape Town Human Research Ethics Committee (REC REF 109/2011).

## Results

### Perceptions of Chronic Disease Management and Policy Knowledge

There was a wide range of opinions as to how this CHC was coping with chronic disease management (CDM). Some felt that it was coping well and had improved, while others felt service delivery was deteriorating. Considering the Western Cape in general, many respondents felt CDM was poor due to poor policy awareness and utilisation of preventative measures, compounded by staffing and time constraints. On the whole, knowledge of the policy was said to be poor, due to unfamiliarity with the policy document or inadequate communication about policy details from management. Many of the clinical staff reported that they had either never heard of or had no training on the policy. Limited policy knowledge was also evident in participant's responses to questions regarding the implementation of specific policy components which are discussed below.

### Perceptions of Practice: Facility-based Systems

Integral to the PGWC Chronic Disease Management Policy is facility-based stabilisation of chronic disease patients interacting seamlessly with community-based maintenance of these patients. Respondents were asked specifically about each aspect of service delivery mentioned in the policy and how these related to this specific CHC. Their views included their perceptions of current practices as well as their perceptions of the factors influencing these practices.

As per the policy outline, the facility-based systems were broken down into a number of different components:

- ○ Service delivery platform (including chronic disease ‘clubs’, equipment and resources)
- ○ CDM Health Care Teams (coordinated by a ‘champion’)
- ○ Clinical governance (including patient satisfaction surveys)
- ○ Education (club-based and therapeutic groups)
- ○ Provision of medication
- ○ Referral
- ○ Outreach and support
- ○ Training
- ○ Information management and research

According to most respondents, a dedicated chronic care team doesn't currently exist and some participants felt that staff were not working as a team to manage chronic diseases at this facility, partly due to a lack of specific practical advice regarding teams in the policy

*“Just one thing about the actual policy is no information of the policy is given on how the chronic team is going to be recruited, trained and established - it just says you must have one. And you know that with all our challenges and our staff constraints, it means it's not going to happen unless some practical advice into the policy is added… it's all about incentives…” (Provincial employee 1)*

As per the policy, a ‘champion’ needs to be nominated to head up this team. There were varying opinions amongst the respondents as to who the champion was at this facility, and various interpretations of the champion's role.

*“I think that the concept of champion is kind of misunderstood…People think that champion is the doer, you know, instead of champion being the person having the knowledge required… and then start spreading and educating…staff to making sure that the chronic disease policy is 100% implemented.” (Clinical manager 3)*

The ‘club’ system exists at this CHC as the service delivery platform for chronic disease management, which means that patients with chronic diseases can be seen separately from the rest of the patients, and with scheduled appointments. Opinions on the club system were mixed, with respondents highlighting both positive and negative aspects.

*“…patients know they can come to this [club] room whenever they need. Sometimes they don't have a booking or they have a sore on their foot - and they feel they can come here because I will make a way that they see a doctor… So they feel like there's a place that they can go if things is not going too well for them… because the connection between the staff and the client… they feel that they won't be like rejected.” (Nurse 1)*

Respondents agreed that the majority of facility-based group education happens in club room while patients are waiting to be seen, since therapeutic groups (consisting of a structured programme of group education sessions given at diagnosis of the chronic disease) don't exist at this CHC at present. Many felt that adequate lifestyle counselling was not being done.

Most respondents indicated that staff lacked clinical competence and insight into certain aspects of chronic disease management, and that training was inadequate. They also felt that staff lacked support, particularly junior staff as well as those who were trying to implement positive changes to the system. Some also felt that there was little or no support from allied services such as dietetics or health promotion.

Respondents were asked to comment about the aspects of chronic disease management that were reported to be both good and poor, according to the Audit Tool. Many felt that there had been some overall improvements. However, others felt that the picture the audit painted was inaccurate as the sample size was too small and gold-standards in management seemed unrealistic.

With regards to other aspects of policy implementation that relate to facility-based care, record keeping, the measurement of patient satisfaction and the provision of support for local community-based services were all identified as areas requiring improvement. In contrast, the provision of centrally pre-packaged medication was believed to have made a significant difference to CDM.

### Perceptions of Practice: Community-based Systems

According to the policy, once patients have been stabilised at the facility they should be down-referred to the community-based services, which encompass NPO involvement, adherence support and community input.

There was uncertainty amongst most of the respondents as to how the community-based support groups (CBSGs) were running and who was running them. Only those who worked directly with these services were able to explain the infrastructure far more clearly: CBSGs are run weekly or monthly by NPOs who are contracted by the provincial government to deliver a very specific package of care.

Referral to these CBSGs for ongoing education and monitoring, once chronic disease patients are stabilized within the facility, is one of the most important aspects of the policy. Facility-community integration is dependent on this pathway being followed. While many were hopeful or determined that it should work, most were unsure if it was actually functioning at present and felt that many staff members were not referring patients to CBSGs.

### Staff-Related Challenges to Chronic Disease Management

Staff-related challenges were among the major problems raised by respondents in terms of chronic disease management, especially considering that chronic disease patients, many with multi-morbidity, make up a significant number of the total number of patients seen at the CHC on a daily basis (documented to be at least 200 patients per day according to PGWC data) [32].

**Figure 2:**
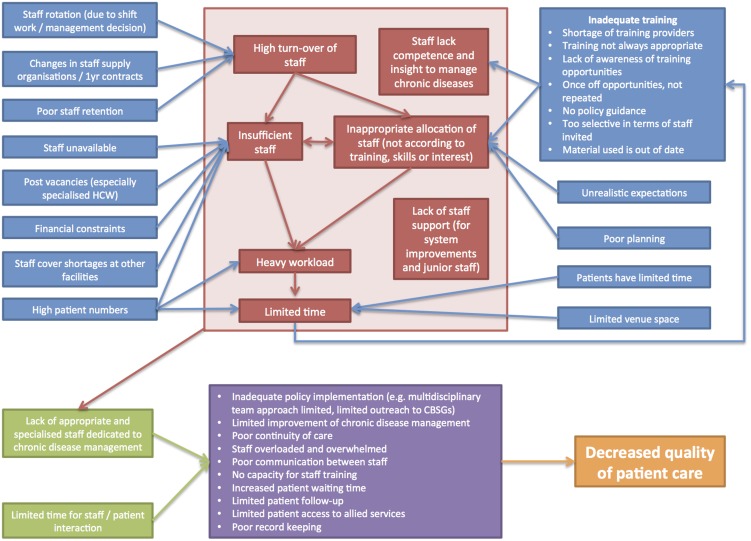
Summary of staff-related challenges.

Staff-related challenges raised by respondents are summarized in figure 2. This diagram broadly demonstrates how a number of different challenges relate to one another: an inadequate number of staff (due to high staff turn-over and post vacancies) exacerbated by high patient numbers, inadequate training and poor staff management results in significant time pressures during consultations as well as a lack of appropriately skilled and specialized staff. The effect of these factors is compounded by poor continuity of care, staff apathy and poor access to allied health services, which ultimately reduces the quality of patient care.

#### Staff Attitudes

Respondents discussed a number of attitudes that they felt existed amongst staff members. Broadly speaking, the dominant attitude described was a sense of apathy amongst staff, which included low motivation and resistance to change. When describing staff attitudes, respondents used words such as: despondent, jaded, disempowered, ambivalent, and overwhelmed. Mixed feelings existed regarding whether it was inappropriate for staff to feel apathetic or whether there were valid reasons as to why they felt apathetic. Some respondents (mostly staff members) commented that these feelings related to external factors within the work environment and that it was difficult to stay motivated, while others (mostly management) felt that staff behaviour itself promoted apathy (‘internal’ factors) and it was therefore within their own power to effect a change in their attitude.

*“…what they [the patients] tell me [is] that staff don't really care… they don't really always specifically use the word ‘rude’, you know, but ‘Hulle worry nie oor my nie, hulle gee nie om nie’ [They're not concerned about me, they don't care] - you know, ‘they don't take note of me’, you know, those kind of things…” (Clinical manager 2)*

Many respondents felt that staff's resistance to change was an obstacle in chronic disease management and policy implementation. Typically this resistance was described to relate to different aspects of the policy, for example, monitoring patients, completing clinical records adequately, limiting investigations, and critically evaluating practices to improve efficiency.

Most respondents expressed negative feelings regarding facility management and felt that managers weren't performing adequately. There appeared to be a disconnect between what staff and management felt to be important in terms of staff allocation. Managers placed greater value on a body of staff with diverse skills (able to work in any clinical domain) whereas staff members expressed a preference for permanent allocation to the specific area they felt passionate about.

#### Staff perceptions of patients

**Figure 3:**
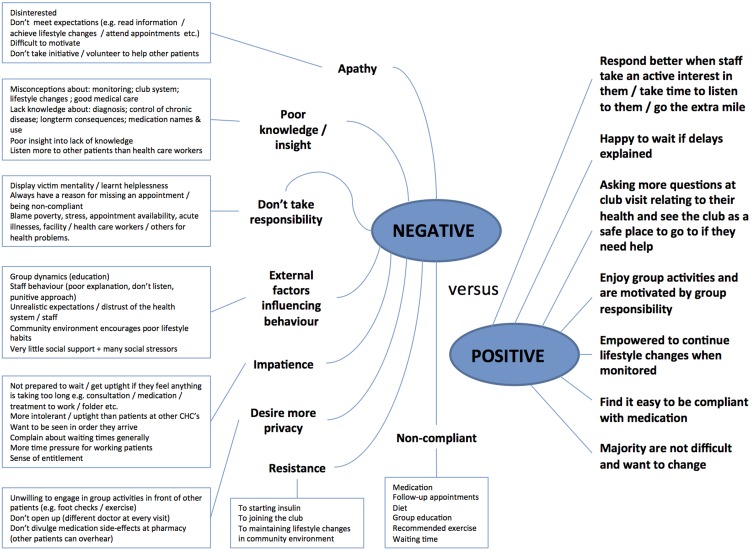
Staff perceptions of patients.

Respondents held a variety of opinions and impressions of patients, mostly relating to patients' knowledge, attitudes and behaviour. These can broadly be divided into negative and positive perceptions and are summarised in a diagram in figure 3. Staff who had negative perceptions of patients mostly spoke about aspects relating to patient apathy whereas staff with more positive perceptions highlighted indications of patient motivation.

*“Although we tell them, okay, fine, diet control and all those things - it seems to me sometimes they want to be pushed for things; they don't want to initiate things themselves…” (Nurse 5)*

## Discussion

The results indicate a gap between policy and its implementation at this facility, which could represent a failure of one or more of the aspects of primary health care: accessibility, continuity, comprehensiveness, coordination and accountability [18]. This highlights a larger gap: the need to strengthen the application of primary health care principles.

One of the most significant reasons for the gap seems to be poor policy knowledge by clinicians who manage chronic disease patients on a daily basis. This is concerning since the poor dissemination of policy [34] and inadequate training [30], [35] have been identified as factors contributing to poor policy implementation. Cheung et al. [36] highlight the need to carefully analyse policy documents to ensure that there is alignment between policy statements and intended outcomes, as well as the importance of policy documents being easily available to those implementing the policy concerned [36]. Further, in a study done in Tanzania on the implementation of new treatment guidelines for Malaria, inadequate training around this new policy was found to be one of the main reasons for poor policy implementation. Poor policy knowledge in this primary care facility meant that no champion had been identified to take ownership of CDM and this seemed to translate into clinicians working individually rather than as a team to manage chronic disease patients. This can result in a fragmented service, with clinicians trying to treat and educate patients on an individual basis with little continuity of care for patients or collaboration between staff members [37], [38]. The factors once again point to gaps in the application of the primary health care approach, with services lacking continuity and comprehensiveness [39].

Intensive group education for patients was not undertaken which, in a busy, overloaded primary health care system, could surely be a time-saving measure. This is supported by unpublished data [K. Manning 2011] on research done in this community. Facility-based staff indicated little or no interaction with community-based services that provide, as one of their services, on-going education and appeared to be well established and functioning effectively. Literature suggests that strong links between internal systems and external services as well as the use of multidisciplinary teams appear to be important requirements in primary health care for successful implementation and sustainability of chronic disease services [39], [40]. However, many patients were not being referred to these services, thereby adding to the facility's patient load which community-based services are designed to relieve [25].

Other major factors that appear to contribute to the gap between policy and its implementation include inadequate numbers of staff and a lack of skill diversity amongst staff members, combined with high patient numbers. Literature suggests that high-quality chronic care delivery was more likely to occur in facilities that were able to sustain smaller patient-physician ratios as well as those which referred patients to integrated community programs [9].

Inadequate financial provision and poor management of the limited finances provided seem to be other barriers to effective policy implementation. This is likely the result of difficulties providing strong, visible, innovative leadership both at a facility and provincial level and highlights the need for the primary health care principle of accountability [20]. Management appear to value a body of staff with diverse skills who are able to work in any clinical domain whereas staff expressed a preference to be permanently allocated to an area they feel passionate about, which could enhance continuity of care.

The results suggest a break-down in relationship between clinical staff and management, as well as local and provincial management staff. There is a general tone of apathy around the ability to affect change, with blame for inadequate chronic disease care being continually shifted from one party to another. Further, negative perceptions around patient behaviour seem to add to the staff's sense of apathy and despondency.

Rispel et al. [30] assessed various aspects of the current South African health system and health policy implementation and highlighted a number of ongoing challenges which were similar to the challenges identified in this study. These included fragmentation of services, poor policy implementation, staffing challenges, resource limitations and little attention to quality of care [30]. Literature also shows that policy implementation difficulties are not limited to chronic disease policy, but exist in other sectors of health care in South Africa as well, such as mental health care services [31], [41].

### Recommendations

The gap between policy and implementation could be bridged in a number of ways. Further, addressing the poor application of primary health care principles across the health care system would also likely result in improved adherence to policy principles.

Firstly, staff need to be better educated around the key tenets of the policy itself, and misconceptions about community-based services need to be addressed. Stronger relationships should be built between facility- and community-based staff. Strong, dynamic, visible leadership which creates a supportive environment is needed to ensure that these changes are made and that existing difficulties are tackled, a recommendation which is supported by current literature [30].

While focusing on improved staff policy knowledge, it might also be important to take a step back in the process, and consider reviewing the policy itself with front-line staff, using a collaborative process to improve staff buy-in and identify practical difficulties. This could be achieved by using a consensus-building method such as nominal group technique [42] and could be the basis of future research.

Participant responses suggest that chronic disease patients would benefit considerably from the presence of dedicated staff for chronic disease management. Based on their comments, it could be recommended that staff members should be permanently allocated and additionally trained to perform only chronic disease care, supported by a dedicated budget. However, current literature does not support the use of a ‘vertical approach’, rather advocating an integrated, patient-centered, team-based and community-oriented primary care approach, also known as ‘horizontal programming’ [43]. Smaller practitioner-patient ratios as well as adequate health budgets are needed if horizontal programming is to be effective, which will then result in health cost saving [9], [19], [29], [44].

Patient education is a key aspect of chronic disease management and should be prioritised at a facility as well as a community level and should be on going to empower patients and their families. However, the approach taken to the delivery of education should be carefully considered; motivational interviewing has been shown to be an effective approach in this regard [45]. Current literature supports the use of a patient-centred approach during consultations [46].

### Limitations and Strengths

Although exploring chronic disease policy implementation at only one facility could be seen as a limitation, this allowed for more in-depth analysis of relevant issues. The relatively small sample size could also be viewed as a limitation of this study. Despite these limitations, the findings of this study could be used to inform future quantitative work with a greater number of primary care facilities. A further limitation is that the principle investigator was contracted to work at the facility as a doctor at the time of data collection. Interpersonal dynamics could have influenced what participants were prepared to share. However, working at the facility meant greater insight into relevant issues as well as a level of familiarity with staff members than an outsider would not have had.

Due to the complexity of the policy document, the guide questions for the focus groups and interviews became quite extensive, which was another limitation. An initial survey to establish the major problems requiring more detailed investigation may have been a way around this. Chronic disease patients were also not included as participants in this study to provide information on the patient-centeredness of care and patient satisfaction. This could be a useful follow-up study.

## Conclusions

Poor policy knowledge seems to be one of the major reasons for the gap between policy and implementation at the facility studied. This results in fragmented individual clinical practice rather than cohesive team work, as well as poor links between facility-based and community-based services. Strengthening leadership at a managerial level could assist with addressing the breakdown in staff-management relationships which adds to staff apathy and contributes to reduced quality in patient care. These issues need to be urgently dealt with in order for chronic disease management to improve at a local level. On a broader scale, greater adherence to primary health care principles, which have been shown in the literature to result in cost-effective care and improved health outcomes [19], [44], could enhance policy implementation. At a philosophical level, this research highlights the tension between primary health care principles and a diseased-based approach in a primary care setting.
